# Postthoracotomy Pain Syndrome Following Surgery for Lung Cancer: Symptoms and Impact on Quality of Life

**DOI:** 10.6004/jadpro.2015.6.2.4

**Published:** 2015-03-01

**Authors:** Kathleen G. Hopkins, Leslie A. Hoffman, Annette De Vito Dabbs, Peter F. Ferson, Linda King, Linda A. Dudjak, Thomas G. Zullo, Margaret Q. Rosenzweig

**Affiliations:** 1 Carlow University College of Health and Wellness, Department of Nursing, Pittsburgh, Pennsylvania;; 2 University of Pittsburgh School of Nursing, Pittsburgh, Pennsylvania;; 3 University of Pittsburgh School of Medicine, Department of Cardiothoracic Surgery, Pittsburgh, Pennsylvania;; 4 University of Pittsburgh School of Medicine, Department of General Internal Medicine, University of Pittsburgh Medical Center Health System, Pittsburgh, Pennsylvania

## Abstract

Postthoracotomy pain syndrome (PTPS) is a common complication following thoracic surgery. Most studies examining the influence of PTPS on patient-reported symptoms include few patients managed using a minimally invasive approach. Associated sensory changes, potentially neuropathic in origin, are not well described. We therefore examined the symptoms and quality of life (QOL) of patients with and without PTPS who underwent a standard thoracotomy (n = 43) or minimally invasive surgery (n = 54). Patients in this prospective, cross-sectional study completed questionnaires to assess pain (McGill Pain Questionnaire), neuropathic symptoms (Neuropathic Symptom Questionnaire), symptom distress (Symptom Distress Scale), anxiety and depression (Hospital Anxiety and Depression Scale), and QOL (Functional Assessment Cancer Therapy–Lung). Excepting younger age (*p* = .009), no demographic or surgical characteristic differentiated patients with and without PTPS. Patients with PTPS described discomfort as pain only (15.1%), neuropathic symptoms only (30.2%) or pain and neuropathic symptoms (54.7%). Scores differed between patients with and without PTPS for symptom distress (*p* < .001), anxiety and depression (*p* < .001), and QOL (*p* = .009), with higher distress associated with PTPS. Despite new surgical techniques, PTPS remains common and results in considerable distress. A focused assessment is needed to identify all experiencing this condition, with referral to pain management specialists if symptoms persist.

Postthoracotomy pain syndrome (PTPS) is defined as pain that develops after a surgical intervention, lasts longer than 2 months, and cannot be attributed to any other cause or condition (International Association for the Study of Pain [IASP], 2011). Following thoracic surgery, the reported incidence of PTPS ranges from 5% to 65%, with 10% of patients reporting severe pain, defined as a > 5 rating on a 10-point scale (IASP, 2011). First reported in 1944 as a consequence of "war wounds of the chest" (Blades & Dugan, 1944), PTPS received limited attention until a seminal study reported the presence of postsurgical pain in a series of 56 disease-free patients up to 5 years after thoracotomy (Dajczman, Gordon, Kreisman, & Wolkove, 1991).

Notably, not all patients who undergo thoracic surgery for lung cancer develop PTPS. The pathology of PTPS has been attributed to rib (Bayram, Ozcan, Kaya, & Gebitekin, 2011; Landreneau et al., 1994), nerve (Bayram et al., 2011; Benedetti et al., 1998; Miyazaki et al., 2011), or muscle (Frola, Serrano, Cantoni, Turtulici, & Loria, 1995; Karasaki et al., 2009) damage from surgery or a chronic pain syndrome initiated by inadequate pain relief in the postoperative period (Demmy, 2009; Dualé et al., 2009). Other potential mechanisms include nerve or muscle damage related to the insertion of chest tubes and drains (Grosen, Petersen, Pfeiffer-Jensen, Hoejsgaar, & Pilegaard, 2012; Guastella et al., 2011; Mongardon et al., 2011). More effective acute and chronic pain management has not been successful in eliminating this condition (Engelhart, Starnes, Hanseman, & Guitron 2014; Grosen et al., 2014), nor has the use of minimally invasive surgical techniques (Wildgaard et al., 2011; Wildgaard et al., 2012). Prior studies provide evidence that minimally invasive surgery is associated with fewer complications in the immediate postoperative period, especially in high-risk and elderly patients (Engelhart et al., 2014; Park, 2011). However, differences in outcomes measured at a longer interval following surgery are less clear.

Although it has been suggested that minimally invasive thoracoscopic procedures may result in less injury and therefore, less risk for PTPS, most prior studies included few (Aoki et al., 2007; Furrer et al., 1997; Karasaki et al., 2009) or no patients managed using a minimally invasive approach (Dualé et al., 2011; Grosen et al., 2012; Guastella et al., 2011; Mongardon et al., 2011; Pluijms, Steegers, Verhagen, Scheffer, & Wilder-Smith, 2006, Sarna et al., 2008). Several prior studies have proposed a neuropathic origin for PTPS (Dualé et al., 2011; Maguire, Ravenscroft, Beggs, & Duffy, 2006; Pluijms et al., 2006; Steegers, Snik, Verhagen, van der Drift, & Wilder-Smith, 2008; Wildgaard et al., 2012). However, there has been limited exploration of this consequence using a battery of standardized instruments to rate symptom distress or impact on quality of life (QOL). The purpose of this study was to describe the symptoms and QOL of patients with and without PTPS who underwent a standard thoracotomy or minimally invasive surgery for lung cancer.

## METHODS

**Design and Sample**

Participants for this prospective, cross-sectional study were recruited during visits to clinics affiliated with a university-based cancer center. Inclusion criteria were as follows: (1) managed surgically for stage I, II, or IIIa lung cancer without evidence of metastasis; (2) between 2 and 12 months postsurgery (conforms to definition of PTPS); and (3) greater than 40 years of age (lung cancer is infrequent in those younger, and if present, likely atypical). Exclusion criteria were as follows: (1) any other cancer diagnosis or metastatic disease (to avoid confounding symptoms); (2) inability to speak, read, or understand English (questionnaires were in English); and (3) presence of comorbidities such as dementia or memory loss (limited ability to participate as informant). A power analysis revealed that 42 participants per group should result in a power slightly over 0.8, with alpha at 0.05. Therefore, a sample of 84 subjects or more was judged to be statistically appropriate. The study was approved by the Institutional Review Board, and all participants provided written informed consent.

A total of 112 potentially eligible subjects were recruited between August 2010 and December 2012. Two patients were not enrolled due to refusal. Of the 110 patients who provided informed consent, 13 did not complete the study for the following reasons: 5 did not return instruments, 5 died, and 3 were no longer eligible due to new metastatic disease. Thus, the final sample consisted of 97 of 110 (88.1%) participants.

All patients were recruited from one surgical service, and practice was similar with regard to operative management and protocols for pain management. Choice of surgical procedure was at the discretion of the operating surgeon. Reasons for performing a thoracotomy vs. a thorascopic procedure included tumor grade, location, lymphovascular invasion, histology type, pleural involvement, size, and surgical margins (Detterbeck, Lewis, Diekemper, Addrizzo-Harris, & Alberts, 2013). A complete surgical resection with curative intent was performed in all cases. No patient received preoperative or postoperative radiation or chemotherapy.

**Measures**

Participants were given six self-report measures that took an average of 30 minutes for them to finish, with the option to complete the instruments in clinic or at home and return them in a preaddressed mailing envelope. Study participants provided informed consent before any completing study instruments.

*Health History Survey (HHS):* A researcher-designed self-report instrument was used to identify personal, social, and medical variations among patients. Personal information was provided by the participant and included age, gender, race, ethnicity, and smoking history. Social information included marital and employment status. Information provided by medical record included comorbidities, tumor type, cancer stage, surgical approach, and surgical procedure.

*Charlson Comorbidity Index (CCI):* The CCI was designed to assess the presence and type of 19 comorbid conditions (Charlson, Pompei, Ales, & MacKenzie, 1987). Each condition included in the medical history was assigned a weight (1 to 6 points) based on the strength of its association with mortality. No weight adjustments were made for age. Instrument reliability and validity have been established in prior testing (Charlson et al., 1987; deGroot, Beckerman, Lankhorst, & Bouter, 2003).

*McGill Pain Questionnaire (MPQ):* This self-report questionnaire was the primary tool used to identify pain resulting from PTPS. It was chosen because it assessed pain intensity (1 to 10 scale), included descriptors that characterized the sensation, and included a figure used to identify specific body locations (McGill, 2009). Of the descriptors, only two ("numb" and "tingling") were used to identify neuropathic symptoms. Because prior studies noted that not just the surgical area but also chest tube and drain sites, were areas of pain, the instructions were modified to request that patients mark all locations of postsurgical pain. Instrument reliability and validity have been established in prior testing (Graham, Bond, Gerkovich, & Cook, 1980; McGill, 2009; Turk, Rudy, & Salovey, 1985).

*Neuropathic Symptom Questionnaire (NSQ):* Because the MPQ was deemed inadequate to appropriately identify symptom descriptors associated with PTPS, the NSQ was added after 51 subjects were recruited. Descriptors included in the NSQ were chosen based on the terminology used by patients during follow-up clinic visits and a literature review (Bousassira & Attal, 2011). When completing the NSQ, participants were asked to "describe their discomfort at the surgical site" and to rate the presence and severity of "tingling," "numbness," "increased sensation due to touch," and "increased sensation due to movement" using a numeric visual scale (NVS), with 0 indicating no discomfort and 10 the worst discomfort possible. McGill Pain Questionnaire descriptors (numb and tingling) were used to identify participants with neuropathic symptoms for subjects enrolled prior to adding the NSQ. With the exception of age (*p* = .016), there were no significant differences in personal, social, or medical characteristics in patients who did or did not complete the NSQ.

*Symptom Distress Scale (SDS):* The SDS was a 13-item, self-report scale designed to assess the subjective distress associated with 11 cancer-related symptoms using a Likert-type scale (1 = least distress to 5 = most distress), with a total score ranging from 13 to 65 (McCorkle & Young, 1978). Higher scores indicate more distress. Ratings were summed to achieve a total symptom score. McCorkle et al. suggested that a total score of 25 to 32 indicated moderate distress and that scores ≥ 33 indicated severe distress (McCorkle & Young, 1978). This total score was the variable used in this study. Instrument reliability and validity of the SDS have been established in prior testing (McCorkle, Cooley, & Shea, 1998; McCorkle & Young, 1978).

*Hospital Anxiety and Depression Scale (HADS):* This instrument was a 14-item questionnaire designed to screen for mood disorders (Zigmong & Snaith, 1983). The HADS was comprised of an anxiety and depression symptom subscale. Each of the subscales contained 7 Likert response items scored 0 to 3, with some scores reversed. The total possible score ranged from 0 to 42. The total possible score for two subscores ranged from 0 to 21. Scores were categorized as normal (range, 0–7), suggestive of a mild mood disorder (range, 8–10), and presence of a reportable mood disorder (range, 11–21). Prior studies have validated use of similar screening tools to evaluate distress in lung cancer patients (Buchanan, Milroy, Baker, Thompson, & Levack, 2010; Carlson, Groff, Maciejewski, & Bultz, 2010). Instrument reliability and validity have been established in prior testing (Bjellend, Dahl, Haug, & Neckelmann, 2002; Snaith, 2003; Snaith & Zigmond, 1994).

*Functional Assessment of Cancer Therapy–Lung (FACT-L):* The FACT-L is a self-report, 44-item questionnaire designed to measure quality of life for lung cancer patients (Cella et al., 1995). The FACT-L is comprised of 5 subscales that measure lung-related symptoms and physical, social, functional, and emotional well-being. Scores for each of the five subscales range from 0 to 28, with higher scores implying higher quality of life. Subscale scores can be summed to calculate a total score (0 to 176; Cella et al., 1995). Instrument reliability and validity have been established in prior testing (Cella et al., 1995; Soni & Cella, 2002; Soni et al., 2002).

**Symptom Categories**

Subjects were first divided into two categories: patients with and without PTPS. No PTPS was defined as an MPQ score of 0 and no neuropathic descriptors. Next, patients with PTPS were divided into three subgroups to assist in exploring the neuropathic components of this condition:

PTPS with pain only: Defined as an MPQ score of greater than 0 with no neuropathic descriptors

PTPS with neuropathic symptoms: Defined as an MPQ score of 0 and one or more neuropathic descriptors

PTPS with pain and neuropathic symptoms: Defined as an MPQ score of greater than 0 and one or more neuropathic descriptors

**Statistical Analysis**

Data analysis was conducted using Statistical Package for the Social Sciences (IBM-SPSS, 2013). Missing data were confined to one subject who did not return the SDS and HADS and a second subject who did not return the FACT-L. Comparisons between participants with and without PTPS were made using chi-square or Fisher’s exact test, as indicated (Pallant, 2007; Tabachnick & Fidell, 2007). The Mann Whitney test was used to test for statistical significance between groups because responses were not normally distributed (Pallant, 2007; Tabachnick & Fidell, 2007). When significant differences were found, post hoc comparisons (Kruskal-Wallis) were performed to detect the point of difference (Tabachnick & Fidell, 2007). Statistical significance was set at p ≤ .05 for all variables.

## RESULTS

**Demographic and Medical Characteristics**

The sample included 97 patients (47 men, 50 women) who ranged in age from 45 to 84 years (mean 67.3 ± 9.7 years); see Table 1. The majority were Caucasian (89; 91.8%), and married or living with a significant other (63; 64.9%). Fifty-nine patients (60.8%) were between 2 and 6 postoperative months and 38 were (39.2%) between 7 and 12 postoperative months. All patients were disease-free at the time of interview, based upon computerized tomography (CT) scans and postsurgical pathology reports. Slightly less than half (43; 44.3%) underwent a thoracotomy, and the remainder (54; 55.7%) underwent a thoracoscopic procedure. PTPS was reported by 53 (54.6%) participants. Patients with PTPS were significantly younger than those without PTPS (*p* = .009). There were no other statistically significant differences.

**Table 1:**
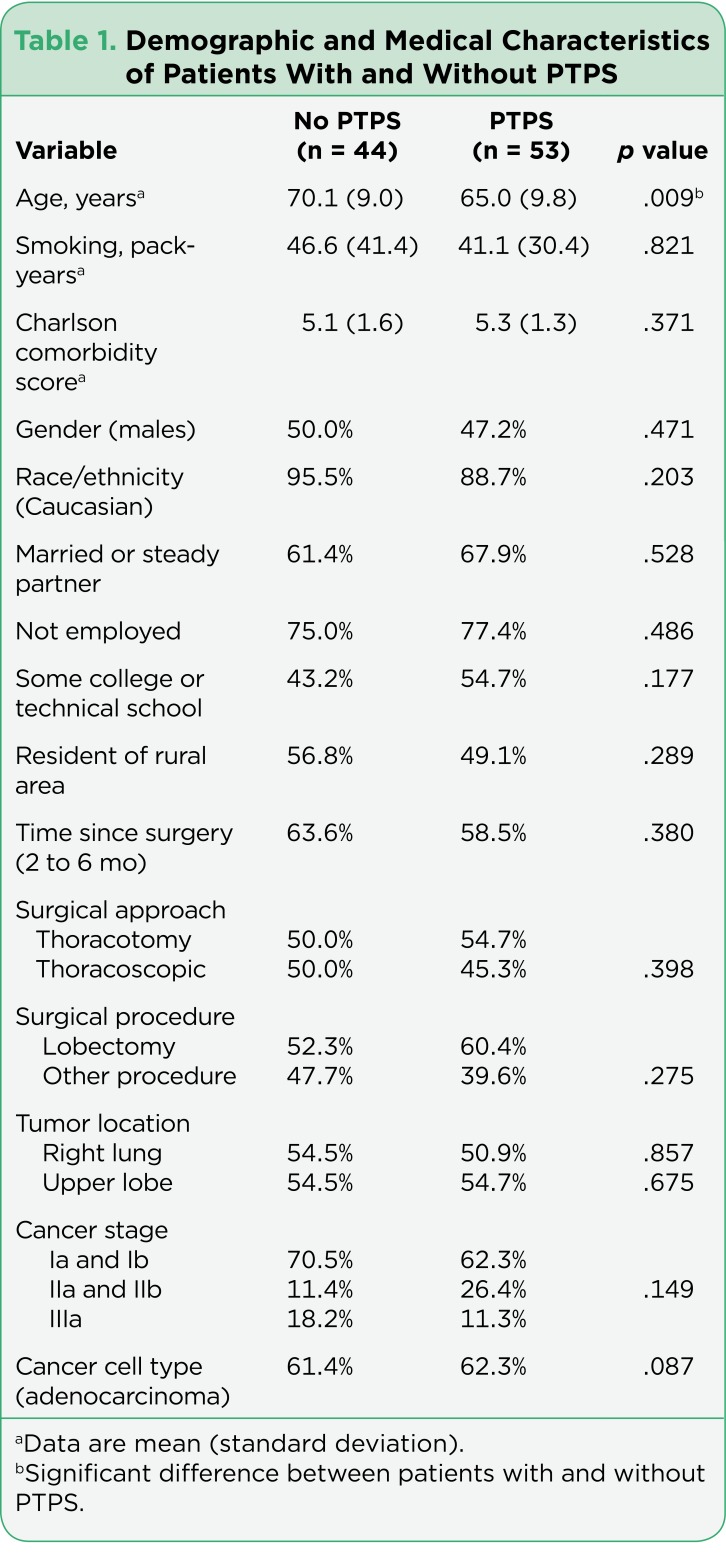
Demographic and Medical Characteristics of Patients With and Without PTPS

**Impact of PTPS**

Patients with PTPS reported a relatively low rating of pain on the MPQ (3.3 ± 3.3; Table 2). Although the majority (60.4%) reported a pain score ≤ 3 (mild pain), 12 (22.6%) reported a score between 4 and 7 (moderate pain) and 9 (17.0%) reported a score > 7 (severe pain). Patients reporting moderate or severe pain were being managed using a variety of medications, including topical solutions, selective serotonin reuptake inhibitors, and opioids.

**Table 2:**
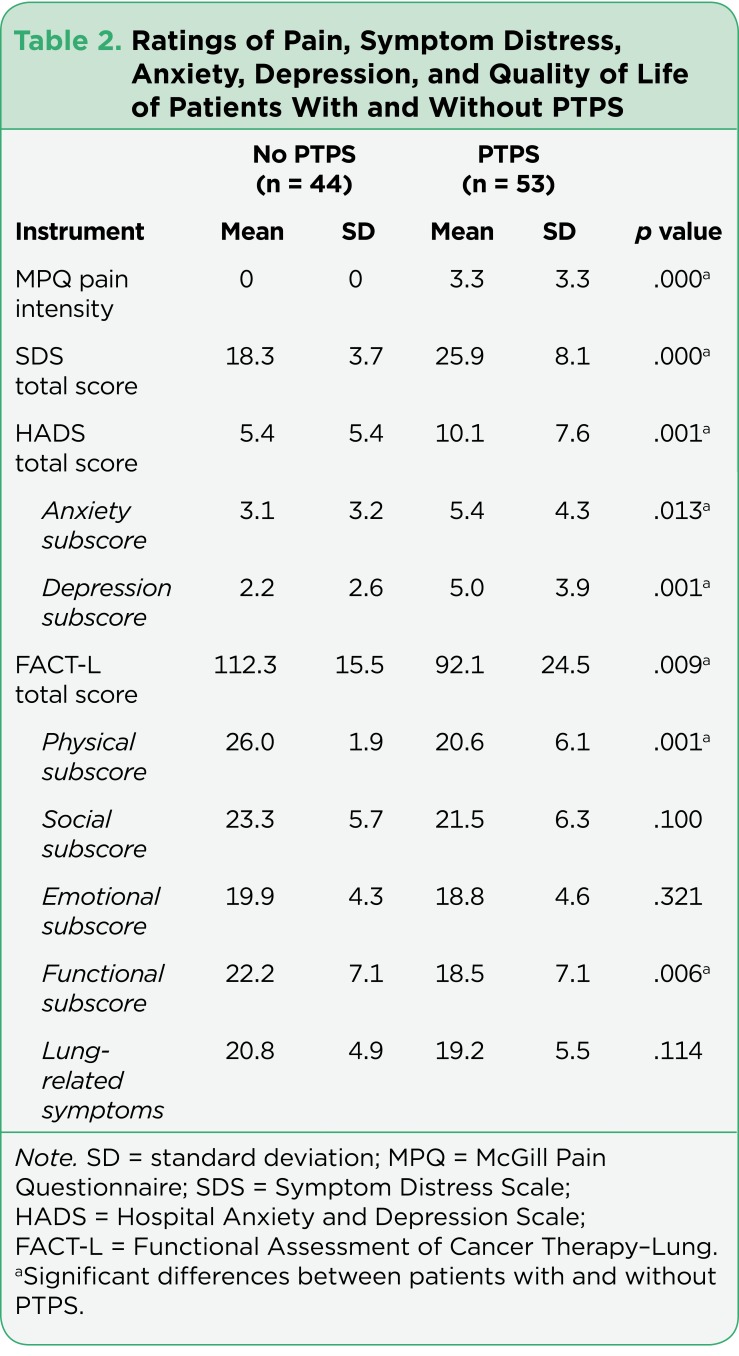
Ratings of Pain, Symptom Distress, Anxiety, Depression, and Quality of Life of Patients With and Without PTPS

Total SDS scores differed between patients with (25.9 ± 8.1) and without PTPS (18.3 ± 3.7), with patients with PTPS reporting significantly (*p* < .001) more distress. Notably, both groups included patients who reported moderate distress (SDS score 25–32). These individuals included 4 (4.1%) patients who reported no symptoms associated with PTPS and 12 (12.4%) patients who reported symptoms associated with PTPS. Ten (10.3%) patients reported scores ≥ 33 (severe distress). All were diagnosed with PTPS and were offered treatment for this condition.

Total HADS scores differed between patients with (10.1 ± 7.6) and without PTPS (5.4 ± 5.4); patients with PTPS reported higher total distress scores (*p* = .001) and higher subscores for anxiety (*p* = .013) and depression (*p* <. 001). Within the total group, 6 (6.2%) subjects reported at least one subscore > 11 for anxiety or depression, which is a reportable level of distress. All were currently under treatment for their symptoms and all were in the group that reported PTPS.

FACT-L total scores differed between patients with (92.1 ± 24.5, range 43 to 136) and without PTPS (112.3 ± 15.5, range 66 to 135). Patients with PTPS reported lower ratings (*p* = .009) for quality of life. Scores for two of the five subscales were significantly different between groups. Patients with PTPS assigned lower ratings to subscores for physical (*p* = .001) and functional (*p* = .006) but not for social, emotional, or lung-related symptoms.

**PTPS Symptom Characteristics**

Of participants with PTPS, 8 (15.1%) reported pain only, 16 (30.2%) neuropathic symptoms only, and the remaining 29 (54.7%) both pain and neuropathic symptoms. With the exception of smoking pack-years, there were no significant differences between groups for any variable examined (Table 3).

**Table 3:**
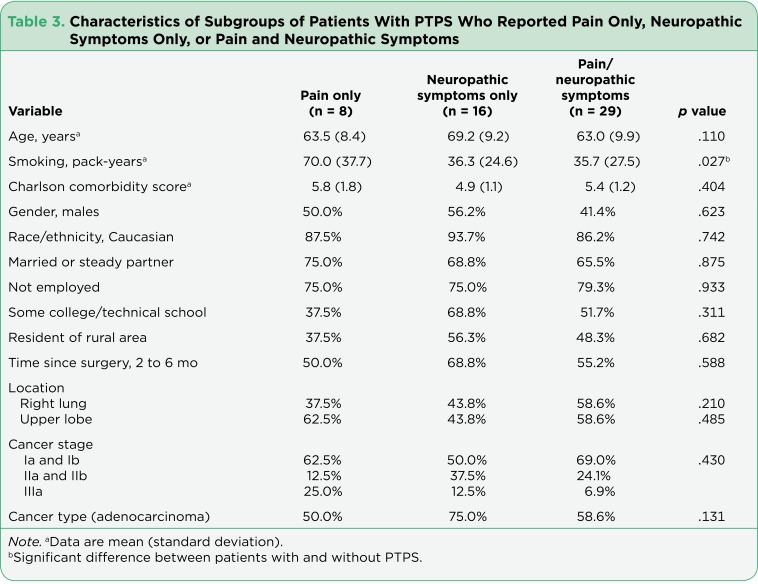
Characteristics of Subgroups of Patients With PTPS Who Reported Pain Only, Neuropathic Symptoms Only, or Pain and Neuropathic Symptoms

**Type of Surgery**

**Figure 1:**
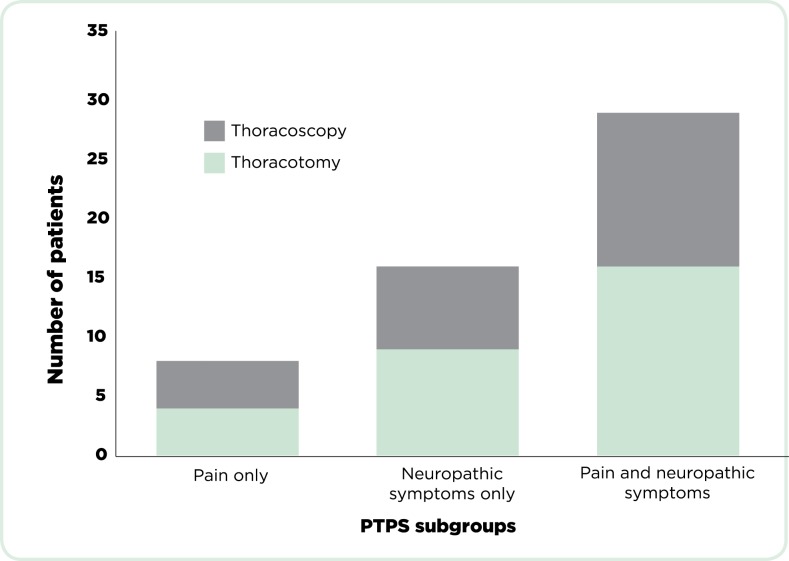
Number of patients who reported pain only, neuropathic symptoms only, or pain and neuropathic symptoms following thoracotomy or thoracoscopy surgery. There were no statistically significant differences between the three groups with respect to surgical approach.

An approximately equal number of patients with PTPS underwent either a thoracotomy (54.7%) or thoracoscopic (45.3%) procedure (Table 1). PTPS participants who underwent both procedures reported pain only, neuropathic symptoms only or pain and neuropathic symptoms (Figure 1). There were no statistically significant differences between the two groups related to type of surgery.

**Location of Discomfort**

**Figure 2:**
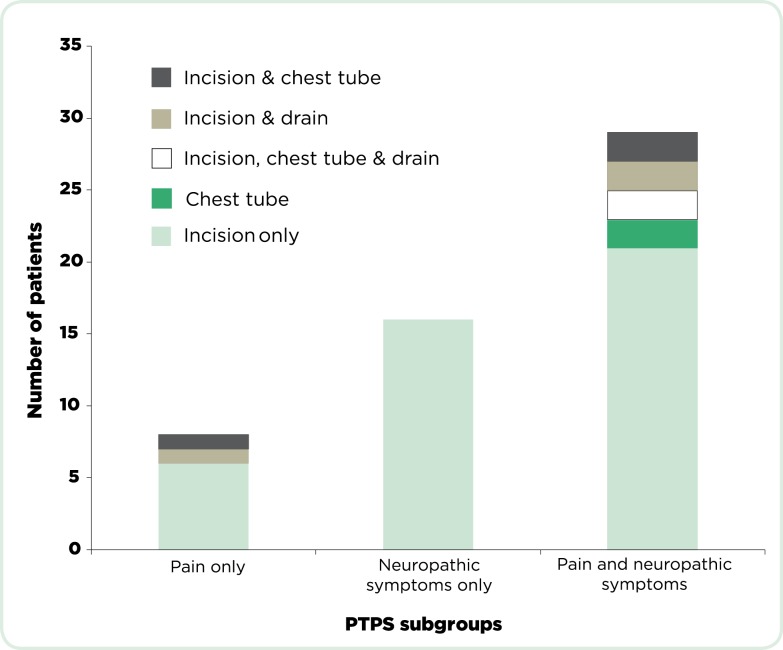
Location of discomfort for patients who reported pain only, neuropathic symptoms only, or pain and neuropathic symptoms. Patients reporting pain only and pain and neuropathic symptoms cited multiple locations. All patients who reported neuropathic symptoms only identified the incision site.

Patients were instructed to mark all location(s) of discomfort on the McGill figure. The eight patients reporting pain only, cited three locations: incision and chest tube, incision and drain, and incision only (Figure 2). There were 16 patients who reported neuropathic symptoms only. All reported discomfort located at the incision site. The 29 remaining participants reported both neuropathic symptoms and pain at various sites.

**Subgroup Ratings of Anxiety, Depressive Symptoms, and QOL**

**Figure 3:**
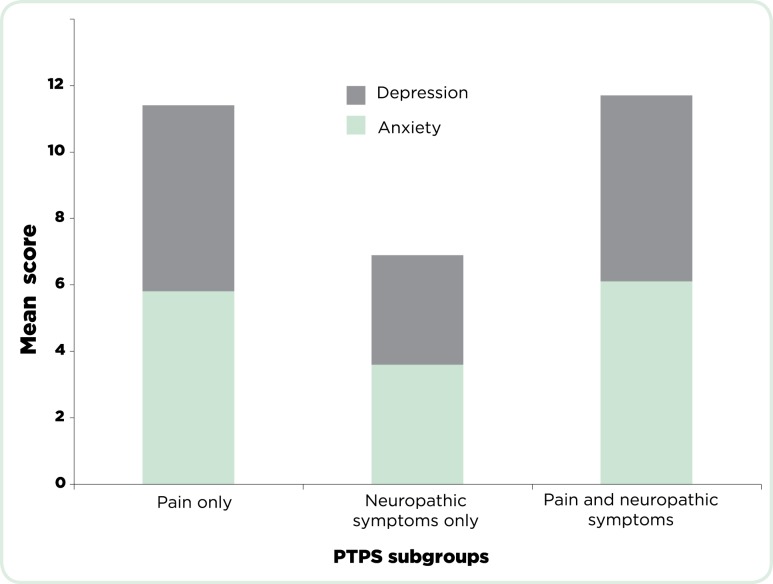
Mean anxiety and depression scores for patients who reported pain only, neuropathic symptoms only, or pain and neuropathic symptoms. There were no statistically significant differences between the subgroups in reports of anxiety or depression.

Because psychosocial experiences can influence PTPS, we also explored the impact of anxiety and depression as determined by the HADS total score and subgroup scores in patients reporting pain only, neuropathic symptoms only, and pain and neuropathic symptoms (Figure 3). There were no statistically significant differences between subgroup scores.

**Figure 4:**
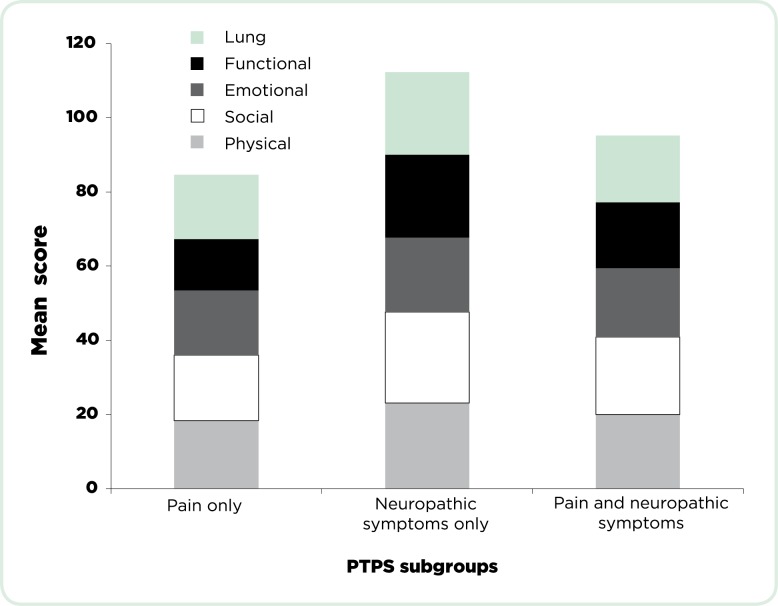
Mean Functional Assessment of Cancer Therapy–Lung (FACT-L) subscores for patients reporting pain only, neuropathic symptoms only, or pain and neuropathic symptoms. The total score for this instrument is the sum of all five of the subscores (lung, functional, emotional, social, and physical). There were statistically significant differences between the FACT-L total scores in patients who reported pain only, neuropathic symptoms only, or pain and neuropathic symptoms. Those individuals who reported neuropathic symptoms only reported higher well-being (*p* = .027) compared with those who experienced either pain only or both pain and neuropathic symptoms.

Impact on QOL was measured by the FACT-L (Figure 4). There were statistically significant differences between FACT-L total scores in patients who reported pain (84.5 ± 28.1; range, 56 to 127), neuropathic symptoms (112.3.1 ± 16.8; range, 79 to 136) or pain and neuropathic symptoms (95.9 ± 24.2; range, 43 to 132). Those individuals who reported neuropathic symptoms only reported higher well-being (*p* = .027) compared to those with pain only or both pain and neuropathic symptoms.

## DISCUSSION

**Major Findings**

There were four major findings in this study: (1) patients who underwent a thoracotomy or thoracoscopic procedure using current surgical techniques did not differ in reports of symptoms consistent with PTPS; (2) patients who experienced PTPS had discomfort at varied locations (incision, shoulder, chest tube, and drain insertion sites); (3) PTPS discomfort manifested as pain only, neuropathic symptoms only, or as a combination of both; and (4) symptom distress and QOL differed significantly in patients with and without PTPS.

**Prevalence of PTPS**

In the present study, which excluded patients with lung cancer metastasis, approximately half (54.6%) of the patients reported symptoms consistent with PTPS when the definition was expanded to include pain, neuropathic symptoms, or both. There was no significant difference in report of symptoms related to the type of surgery (*p* = .382) or time since surgery (*p* = .380).

In 1994, a survey of 343 patients managed at our Center reported no difference in pain 1 year following a thoracotomy or thoracoscopic procedure (Landreneau et al., 1994). Furrer et al. (1997) reported similar findings from a matched study of 30 patients recruited during the same time period. In their study, 33% of patients who underwent a thoracotomy and 36% of patients who underwent a thoracoscopic procedure reported pain or discomfort 3 to 18 months after surgery. More recently, findings from two surveys (Steegers et al., 2008; Wildgaard et al., 2011) that included a total of 750 patients reported a similar prevalence of persistent pain following a thoracotomy (33%–40%) or thoracoscopic procedure (25%–47%) at 22 to 23 months following surgery. From a study that enrolled patients at approximately the same time as the present study, Sarna et al. (2008) reported pain in 24.8% and 22.1% of patients at 2 and 4 months postthoracotomy, respectively. These studies, although conducted at different times historically and different intervals following surgery, report an incidence of PTPS that ranged from 22% to 47%. Our report of a higher incidence (54%) likely reflects our expanded methodology, which asked patients to indicate all sites where they experienced discomfort and a definition of PTPS that included neuropathic symptoms.

To evaluate the contribution of intercostal nerve damage to the development of PTPS, Miyazaki et al. (2011) assessed nerve function using a series of stimuli (2000 Hz, 250 Hz, and 5 Hz) for 24 weeks following surgery for lung cancer. Function of myelinated nerve fibers was significantly impaired following surgery that involved use of rib retractors but absent when these were not used, supporting the notion that these fibers are susceptible to damage by pressure or stretch. Patients managed using video-assisted surgery without metal retractors reported no pain at 12 weeks following surgery. Conversely, approximately 70% of those undergoing video-assisted minithoracotomy with metal retractors and conventional thoracotomy reported pain. Although these findings hold promise as a means to reduce the prevalence of PTPS, there will likely continue to be extensive numbers of patients who experience this condition given the multiple factors that influence surgical decisions, including size of the lesion, ability to localize and remove the tumor, and surgeon preference.

**Location of Symptoms**

Consistent with prior findings, most patients reported pain or symptoms associated with neuropathy at the site of the incision. In addition, chest tube and drain insertion sites and shoulder area were mentioned. Mongardon et al. (2011) reported that 21 (32%) of 65 thoracotomy patients noted more than one painful site, most frequently the incision and chest tube insertion site. Guastella et al. (2011) reported pain localization in an area entirely or largely distributed within the T5/T6 dermatomes on the operated side. Half of their patients described pain in the mammary or submammary area and the remainder in a more diffuse areas, including the sternal/parasternal area and drain insertion point. Grosen et al. (2012) identified sites on the anterior, posterior and lateral chest wall. These findings are important, as they reinforce the need to inquire about pain and discomfort at various sites on the chest wall. In our study, two patients reported pain and neuropathic symptoms that were only present at the chest tube insertion site or shoulder region.

**Symptom Presentation**

We categorized reports of discomfort into three categories: pain only, neuropathic symptoms only, or the combination of both. In our study, most patients 29 (54.7%) identified a combination of symptoms. However, 8 (15.1%) identified pain only and 16 (30.2%) identified neuropathic symptoms only. Prior studies have reported a varying prevalence of neuropathic symptoms. Steegers et al. (2008) used a validated screening tool, the PainDETECT Questionnaire, to assess symptoms in 204 patients. At a median time of 23 months following surgery, 23% were described as having definite neuropathic pain and 30% probable neuropathic pain. Guastella et al. (2011) evaluated 54 patients 6 months after thoracotomy and identified 29% with neuropathic pain and 70% with chronic pain using a symptom grading system and the DN4, a screening tool for neuropathic pain. Mongardon et al. (2011) reported chronic pain in 48% of patients, neuropathic symptoms in 12% and 40% with neither pain nor neuropathic symptoms. These findings appear similar to ours, although comparison is difficult due to differences in study methodology.

Several validated questionnaires are available for use in detecting the prevalence of neuropathic symptoms and describing related characteristics, including the DN4 and PainDETECT (Bennett et al., 2007; Bousassira & Attal, 2011; Freynhagen, Baron, Gockel, & Tolle, 2006) and, because of psychometric testing, may be more appropriate to serially monitor distress from PTPS than the NSQ. Serial monitoring using these validated instruments is strongly recommended to permit comparison between centers in regard to prevalence of PTPS. In addition, there appear to be differences in the ability to detect changes in tactile and thermal stimuli as well as side-to-side symmetry in patients with and without PTPS (Wildgaard et al., 2012). Further assessment of these differences before and after surgery may yield beneficial insights into causes of this syndrome.

**Symptom Distress and Impact on QOL**

Although pain was a frequent complaint, the majority of patients identified pain as mild with mean ratings in the range of 3.3 ± 3.3. However, a substantial minority reported moderate (22.6%) or severe (17.0%) pain, consistent with findings from prior studies (Grosen et al., 2012; Guastella et al., 2011; Wildgaard et al., 2011). Using standardized instruments, we also found significant differences between groups of differences in patients with and without PTPS in regard to symptom distress, presence of anxiety and depressive symptoms, and QOL. All instruments used in this study to monitor these aspects of patient response were brief and, in our experience, required approximately 20 to 30 minutes to complete if all were utilized. Serial monitoring of symptom distress using standardized instruments, including presurgical baseline measurement, is highly recommended to elicit objective data regarding the contribution of preexisting risk factors and response to various therapeutic initiatives. Prior studies support high levels of symptom distress in patients diagnosed with cancer (Buchanan et al., 2010; Carlson et al., 2010) that can be influenced by a variety of factors, including time of surgery (Lehto, 2011), coping style (Prasertsri, Holden, Keefe, & Wilkie, 2011), and treatment (Shimizu et al., 2012). One large study of 1,334 lung cancer patients reported that 12.4% reported depressive symptoms based on HADS subscale scores (Shimizu et al., 2012). Hence, it is important to assess symptom distress at baseline and serially over time.

## LIMITATIONS

Our study used a cross-sectional design that limited assessment of symptoms to a single time point. It is possible that symptoms may have differed over time. However, we found no difference in the number of patients reporting symptoms of PTPS based on time since surgery. The sample was recruited from a high-volume academic service specializing in thoracic surgery. Results may not be generalizable to other practice settings. Approximately half of the subjects did not complete the NSQ, as it was added midstudy. MPQ descriptors ("numb" and "tingling") were used prior to adding the NSQ. Patients with PTPS or subgroups may have been over or underestimated using this approach. Finally, we did not distinguish between muscle sparing and open thoracotomy nor did we distinguish between video-assisted and robotic thoracoscopic surgeries.

## CLINICAL IMPLICATIONS

Despite new innovations in surgical technique, PTPS remains common. Advanced practitioners managing the care of these patients need to be aware of the various ways symptoms can manifest, i.e., pain only, neuropathic symptoms only or a combination of these factors in various body locations, and question patients specifically regarding their presence. Referral to specialists in pain management should be considered if initial interventions prove ineffective in achieving symptom relief (Wildgaard et al., 2011; Wildgaard et al., 2012). A targeted physical assessment (Hopkins & Rosenzweig, 2012) and/or brief questionnaires can guide evaluation of response to therapy (Bennett et al., 2007; Bousassira & Attal, 2011; Freynhagen et al., 2006; McGill, 2009).

Patients may not share their symptom profile unless directly questioned and often attribute all symptoms to a return of cancer. Serial assessment of physical and psychological sequelae insures comprehensive evaluation of symptoms and concerns and provides objective data that can be used to guide management. Future studies should focus on identifying best treatment approaches to manage the complex and varying symptoms seen in this patient population.
